# Supplementary figures and images for: Genetically manipulating endogenous Kras levels and oncogenic mutations in vivo influences tissue patterning of murine tumorigenesis
Source: eLife. 2022 Sep 7;11:e75715. doi: 10.7554/eLife.75715 (PMC9451540; doi:10.7554/eLife.75715)

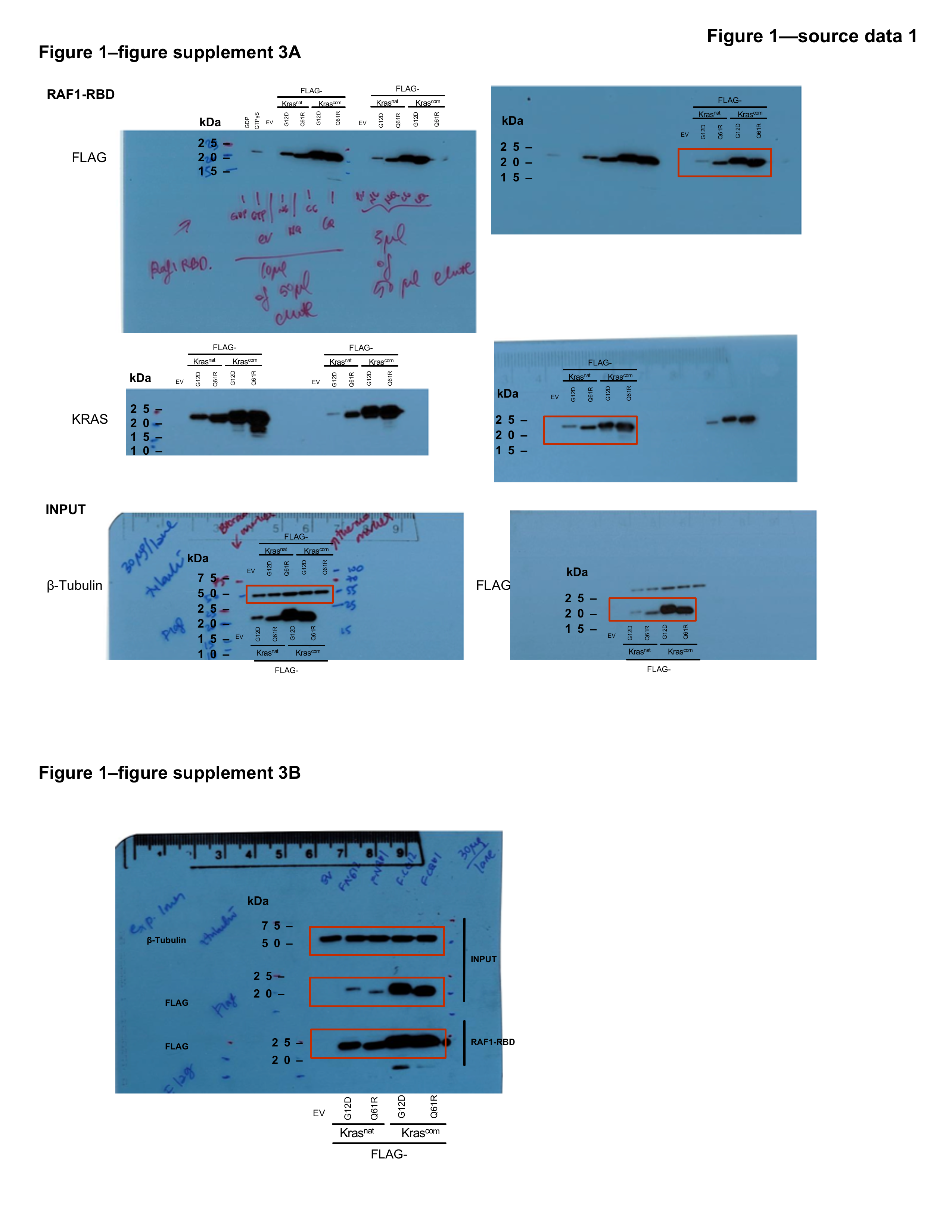

Supplement: Figure 1—source data 1. — Full-length gel images from RAF1-RBD pull downs (top) and whole-cell lysates (bottom) from HEK-HTs ectopically expressing engineered Kras constructs shown in Figure 1—figure supplement 2. [file elife-75715-fig1-data1.png]

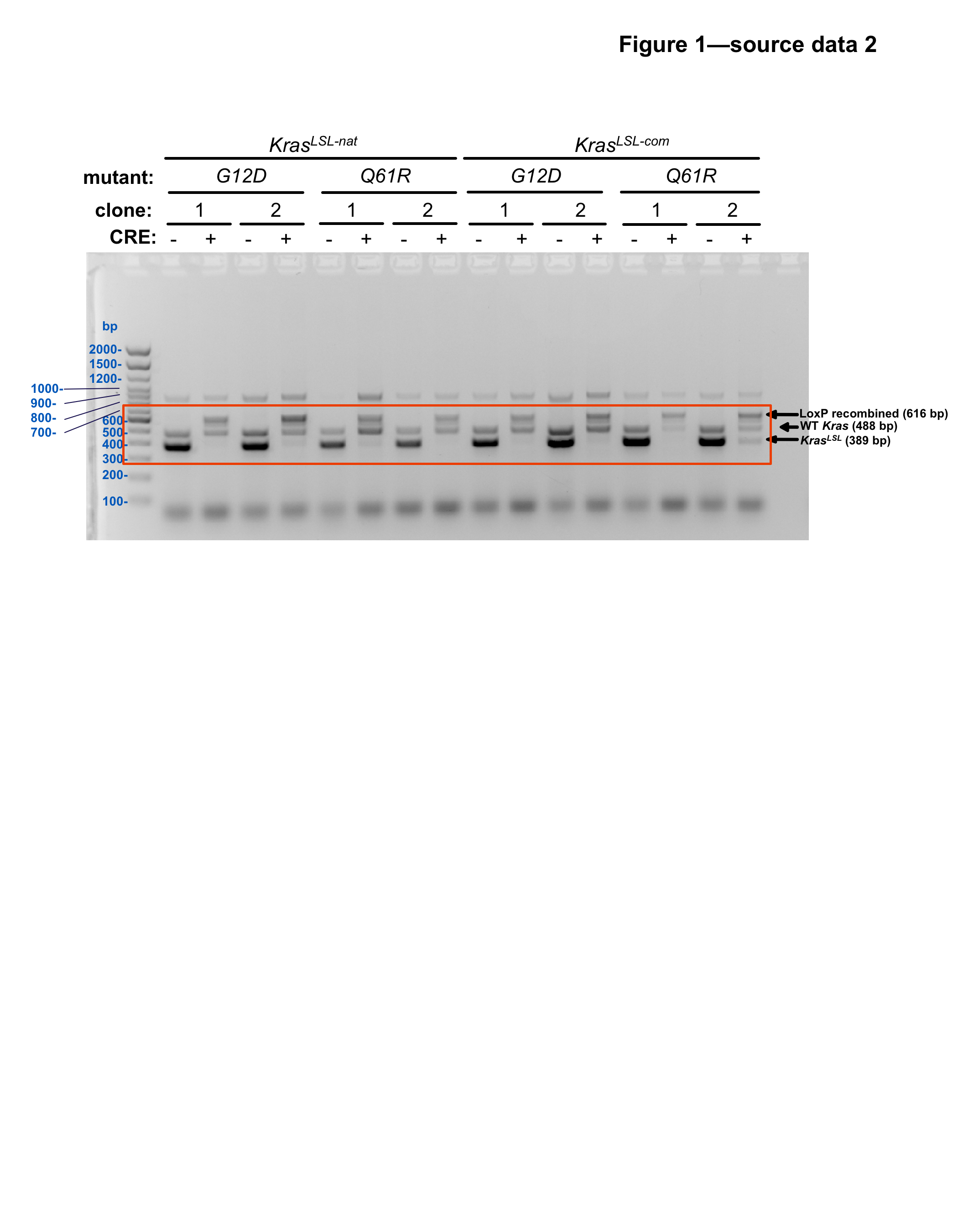

Supplement: Figure 1—source data 2. — PCR genotyping of two independently derived MEF cultures with the indicated KrasLSL alleles in the absence and presence of Cre recombinase (CRE) to detect the unaltered wild-type Kras allele product (WT, 488 bp) as well as the unrecombined (KrasLSL, 389 bp) and recombined (LoxP recombined, 616 bp) Kras allelic products. Gel images were color inverted for better visualization. Red box depicts region shown in Figure 1B. [file elife-75715-fig1-data2.zip › F1 SD2.png]

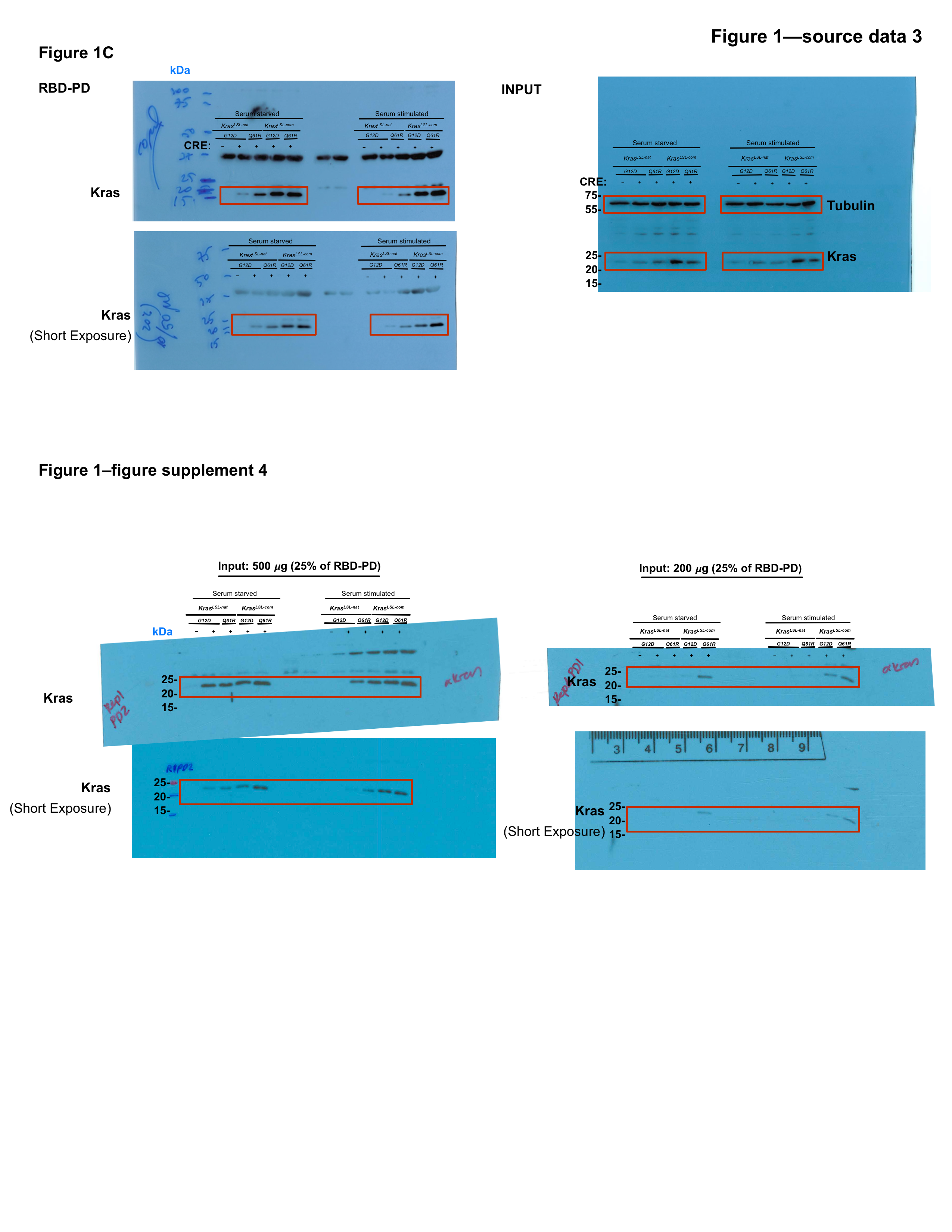

Supplement: Figure 1—source data 3. — Full-length gel images from RBD pull-downs (left) and whole-cell lysates (right) from MEFs derived from KrasLSL alleles with serum starvation, or serum starvation followed by serum stimulation shown in Figure 1C, and same conditions with a second clone of MEF cultures with serial dilutions of 500 μg lysate (left) and 200 μg lysate (right) used for RBD-PD as shown in Figure 1—figure supplement 3. Red box depicts regions shown in Figure 1C and Figure 1—figure supplement 3. [file elife-75715-fig1-data3.png]

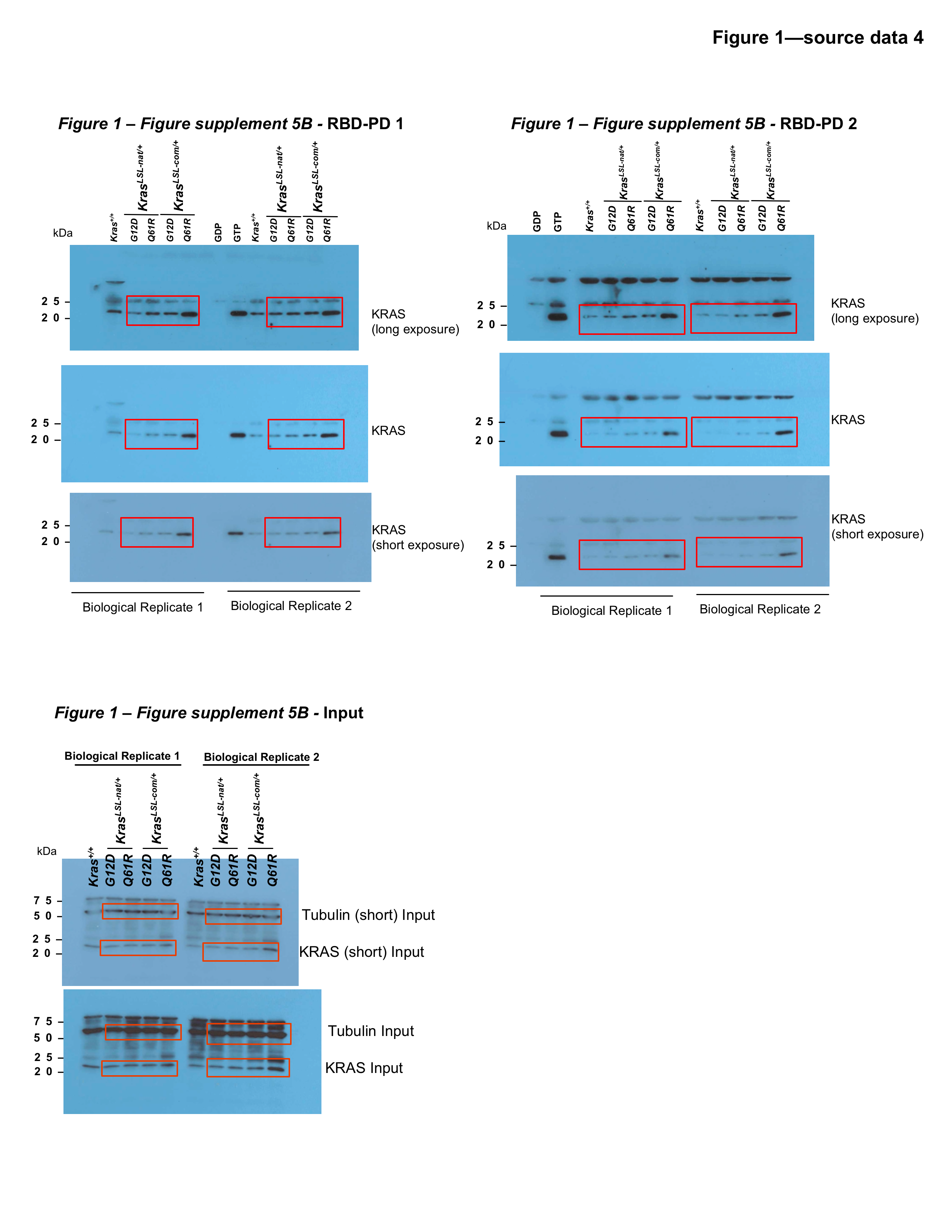

Supplement: Figure 1—source data 4. — Full-length gel images from RAF1-RBD pull-downs (RBD-PD, top) and whole-cell lysates (bottom) of lungs from mice with KrasLSL alleles seven days after tamoxifen injection as shown in Figure 1—figure supplement 4A. Immunoblots of two separate pull downs from two biological replicates are shown as in Figure 1—figure supplement 4B. Red box depicts the regions shown in Figure 1—figure supplement 4B. [file elife-75715-fig1-data4.png]

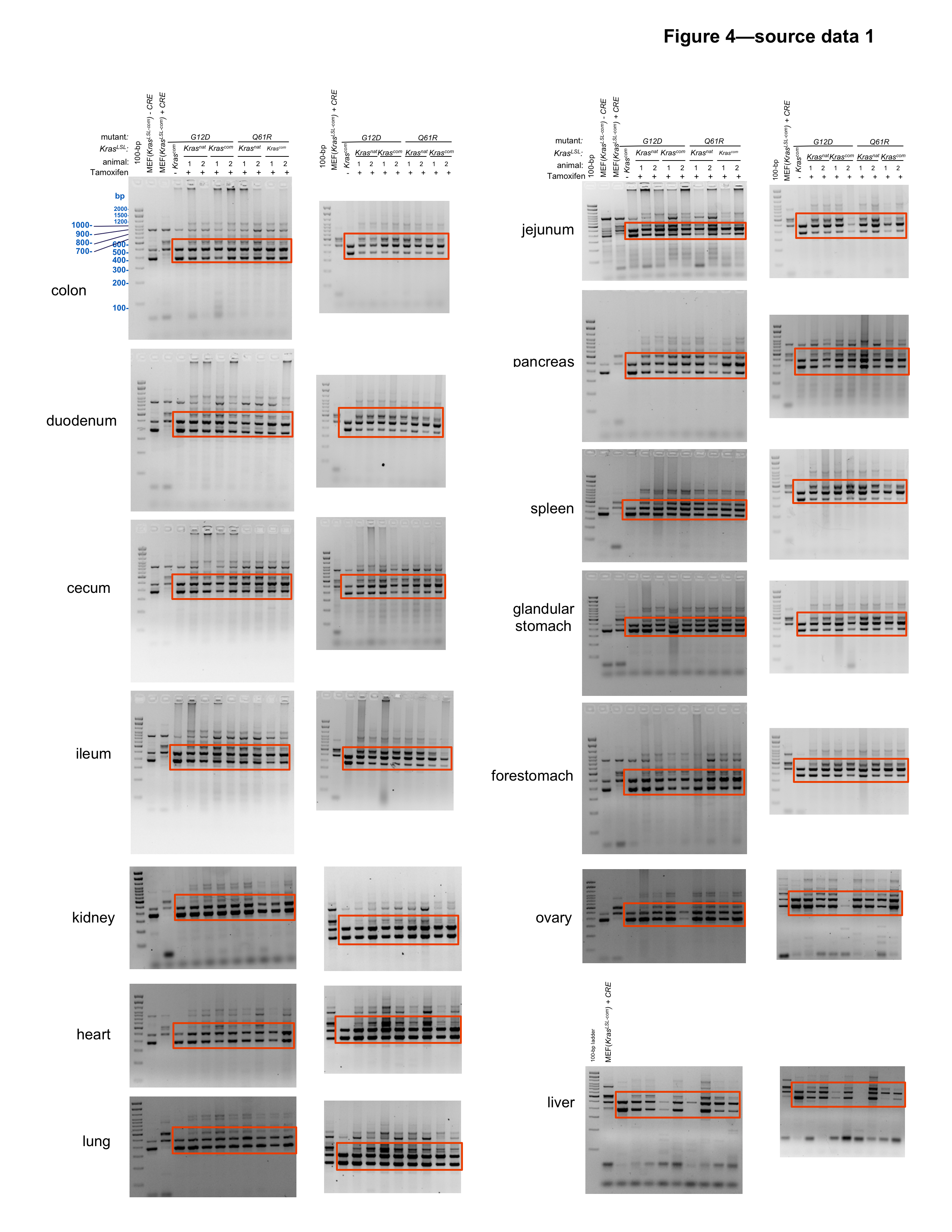

Supplement: Figure 4—source data 1. [file elife-75715-fig4-data1.zip › F4 SD1.png]
